# A dataset on the socioeconomic and behavioural impacts in Sri Lanka through multiple waves of COVID-19

**DOI:** 10.1016/j.dib.2024.110063

**Published:** 2024-01-15

**Authors:** Gayanthi A. Ilangarathna, Lakshitha Ramanayake, Neranjan Senarath, Yasiru Ranasinghe, Harshana Weligampola, Wathsala Dedunupitiya, Isuru Thilakasiri, Roshan Godaliyadda, Parakrama Ekanayake, Vijitha Herath, Janaka Ekanayake, Sakunthala Yatigammana, Anuruddhika Rathnayake, Mallika Pinnawala, Muthucumaru Maheswaran, Ganga Thilakaratne, Samath Dharmarathne

**Affiliations:** a Faculty of Engineering, University of Peradeniya, Peradeniya 20400, Sri Lanka; b Department of Geography, Virginia Polytechnic Institute and State University, Blacksburg 24060, United States; c Whiting School of Engineering, Johns Hopkins University, Baltimore 21218, United States; d Department of Electrical and Computer Engineering, Purdue University, West Lafayette 47907, United States; e Faculty of Arts, University of Peradeniya, Peradeniya 20400, Sri Lanka; f Faculty of Medicine, University of Peradeniya, Peradeniya 20400, Sri Lanka; g Department of Electrical and Computer Engineering, McGill University, 845 Sherbrooke Street, West Montreal, Quebec, Canada; h Institute of Policy Studies, Colombo 00700, Sri Lanka

**Keywords:** COVID-19, Socioeconomic impact, Educational Impact, Impact of COVID-19, Household Survey, Door-to-door survey

## Abstract

The impact of the COVID-19 pandemic was diverse and disproportionate among nations, and population segments. The impacts of the disease and the containment strategies adopted are broad and cut across multiple facets of life, society, and the economy, which are intimately interlinked. Therefore, a large household survey was conducted to ascertain the socioeconomic impact and human behavior changes due to the pandemic and the containment strategies covering all provinces of Sri Lanka. The ramifications on mobility and human behavior, income, economic status, food consumption, education, access to health services and information, and cultural and psychological changes were explored, and the data are reported in this paper. The survey was conducted on 3020 households, selected using a multistage clustering technique, to assess the impacts of the pandemic through three distinctly identified waves/phases of the pandemic in Sri Lanka. This dataset will enable researchers and policymakers to analyze the impact of the pandemic through a multifaceted perspective enabling a more holistic approach to decision-making.

Specifications Table

**Table:** 

Subject	Social Sciences
Specific subject area	Social Science, Education, Health, Human Factors and Ergonomics, Media Technology
Data format	Analyzed, Filtered
Type of data	Table
Data collection	We conducted a cross-sectional Household survey covering all 9 provinces including 20 districts in Sri Lanka from August 2021 to September 2021. This dataset consists of data collected from 3020 households, on the impact of the pandemic through three distinctly identified pandemic waves in Sri Lanka. The questionnaire was designed to capture COVID-19 impact in 2 primary sections (socioeconomic impact and behavioural impact) which were further divided into 8 sub-sections: educational impact, mobility impact, access to health services, economic impact, human interactions, food consumption, religious and cultural and psychological impact.
Data source location	Institution (Data Storage Location): University of Peradeniya, Peradeniya 20400, Sri Lanka Districts (Data Collection Locations): Ampara, Anuradhapura, Badulla, Batticaloa, Colombo, Galle, Gampaha, Jaffna, Kalutara, Kandy, Kegalle, Kurunegala, Matale, Matara, Monaragala, Nuwara Eliya, Polonnaruwa, Puttalam, Rathnapura, Vavuniya Country: Sri Lanka
Data accessibility	The dataset is uploaded on Harvard Dataverse Repository name: Socioeconomic Impact of COVID-19 during three pandemic waves Data identification number: https://doi.org/10.7910/DVN/CXMJSM Direct URL to data: https://doi.org/10.7910/DVN/CXMJSM

## 1 Value of the Data

• Contains data from diverse groups of people with different socioeconomic backgrounds, occupations, income levels, education levels, and ethnic identities and serves as a source to identify the groups of people who were disproportionately affected by the pandemic in underdeveloped nations.
• Contains different impact sections and 283 impact-related variables/features for 3020 households scattered around Sri Lanka covering all 9 provinces.
• Can be used to analyze the progression of these impacts on diversely identified groups of people as the pandemic lingered on and their progression through the multiple phases of the pandemic.
• Gauges the effectiveness or responsivity of the population to the enforced containment strategies and their evolution as the pandemic progressed.
• Is anticipated to help policymakers and researchers analyze the impact of the pandemic through a multidisciplinary perspective enabling a more holistic approach to decision-making.
• Opens a gateway to explore the correlations and dependencies between the socioeconomic state and the behavior of the citizens.

## 2 Data Description

Each row of the Data file 1 & 2 represents a single household, and each column represents a variable/feature. Data file 1 (Demographics.xlsx) has the demographic information for each household. The description of variables and the coding used can be found in the Data file 3 (Variable definition and coding system.xlsx). Table 1 provides an overview of the available data files. The full set of questions used in the survey can be found in Data file 4 (Questionnaire_English.pdf) and detailed descriptions about the levels of relevance is also included in the file. A detailed description of the impact areas covered in the survey questionnaire and their subsections are tabulated in Table 2 and it illustrates the different angles at which the survey questions were formed to capture the most information possible.

**Table 1:** Overview of data files/data sets.

Label	Name of data file/data set	File types (File extension)	Data repository and identifier (DOI or accession number)
Data file 1	Demographics.xlsx	MS Excel file (.xlsx)	HARVARD Dataverse (https://doi.org/10.7910/DVN/CXMJSM) [1]
Data file 2	Socieconomic_Impact_Dataset.xlsx	MS Excel file (.xlsx)	HARVARD Dataverse (https://doi.org/10.7910/DVN/CXMJSM) [1]
Data file 3	Variable definition and coding system.xlsx	MS Excel file (.xlsx)	HARVARD Dataverse (https://doi.org/10.7910/DVN/CXMJSM) [1]
Data file 4	Questionnaire_English.pdf	PDF file (.pdf)	HARVARD Dataverse (https://doi.org/10.7910/DVN/CXMJSM) [1]
Data file 5	Data_Description.pdf	PDF file (.pdf)	HARVARD Dataverse (https://doi.org/10.7910/DVN/CXMJSM) [1]

**Table 2:** The main impact sections and the sub-sections of the questionnaire.

Section	Subsection	Description
Demographic Information	Gender	• The basic demographic information of the main respondent is recorded.
	Age	
	Marital Status	
	Education Level	
	Ethnicity	
	Employment Status	
	Employment Sector	
Impact on Education	Changes in the education pattern	• Information regarding the frequency of attendance of respondents to online and in-class education. • Amount of educational work carried out in comparison with pre-pandemic. • New learnings • Preference between online and in-person classes • The devices used
	Access to resources	• Device ownership and capability to purchase devices. • Interruptions due to connectivity issues, power failures, and device malfunctions. • The technical know-how • Package limitations.
	Feelings about self-directed learning at home	• Feelings on, **–** Lacking social aspects of in person education. **–** The Learning Environment. **–** Availability of human resources and support.
Impact on access to health services and information	Access to health services	• Ability to attend health clinics and access doctors with reasoning. • Availability of medicine. • Receival of health guidelines
	Information regarding the pandemic	• The feeling about the reliability of the received pandemic related information. • The sources of information.
Impact on Income and Economic Status	Impact on livelihood and household income	• Changes in employment routine. • Changes in income. • Additional Measures taken for compensation.
	Financial supports during the identified periods.	• Receival of financial assistance and other benefits from the government and other organizations.
	Change in food consumption during identified pandemic phases compared to the pre-pandemic period.	• Availability of food in terms of quantity. • Reasons for increased or decreased consumption.
Impact on Mobility	Frequency of accessing food sources	• An assessment of the modes/ways used to fulfill the food needs are explored. • The respondents were questioned on how often they visited nearby shops, the main supermarket in the city, a mini supermarket nearby, home delivery, their own garden, government/NGOs, online apps, from neighbors, fairs, and others to collect supplies needed.
	Frequency and methods of accessing financial services	• Study the localities to access financial services during the pandemic, including banks located in town/city, banks located nearby, Local financial Institutes, ATMs in the city, ATMs nearby, Online banking, Post offices, and other.
	Frequency of access to the leisure activities	• How often people participated in outdoor activities such as going on trips, visiting recreational places, Social Gatherings, and other places during a pandemic.
	Frequency of using modes of transportation	• An exploration to identify the dominant modes of transportation including public transport, shared vehicles, Use of hired three-wheeler, use own motor vehicles, Use of bicycles, Walking and other methods during COVID-19.
	Frequency of residing at following places to attend for work.	• Study to analyze the way/mode that people used to attend work. (Work from home, come to work from home, come to work from rented out place/ boarding place, come to work from a common hostel, or Other)
	Frequency of using the following mode of transportation for work	• Study the likelihood of using different transportation methods to report their workstations including the Use of public or Shared vehicles (School vans, staff vehicles, private vehicles provided by the institute, Use of hired three-wheelers, Use of own motor vehicles, Use of bicycles, Walking, Other).
	Frequency of experiencing the levels of interaction in work	• How often level of people interacted in their workplace, Work alone at home, Work with few known groups of people, Work with regular few known people and limited unknown people, Work with irregular few unknown people, Work with irregular large groups, Other.
Psychological Impact	Effect of lockdown on one's emotions, feelings, and various aspects of life	• How respondents feel about the pandemic and lockdown periods.
	Effect of lockdown on relationships	• How the pandemic affected relationships among family members, neighbors, and office peers
Impact on Cultural behavior	Impact on cultural engagements and behaviors	• How the pandemic affected cultural beliefs and engagements.

**Fig. 1:**
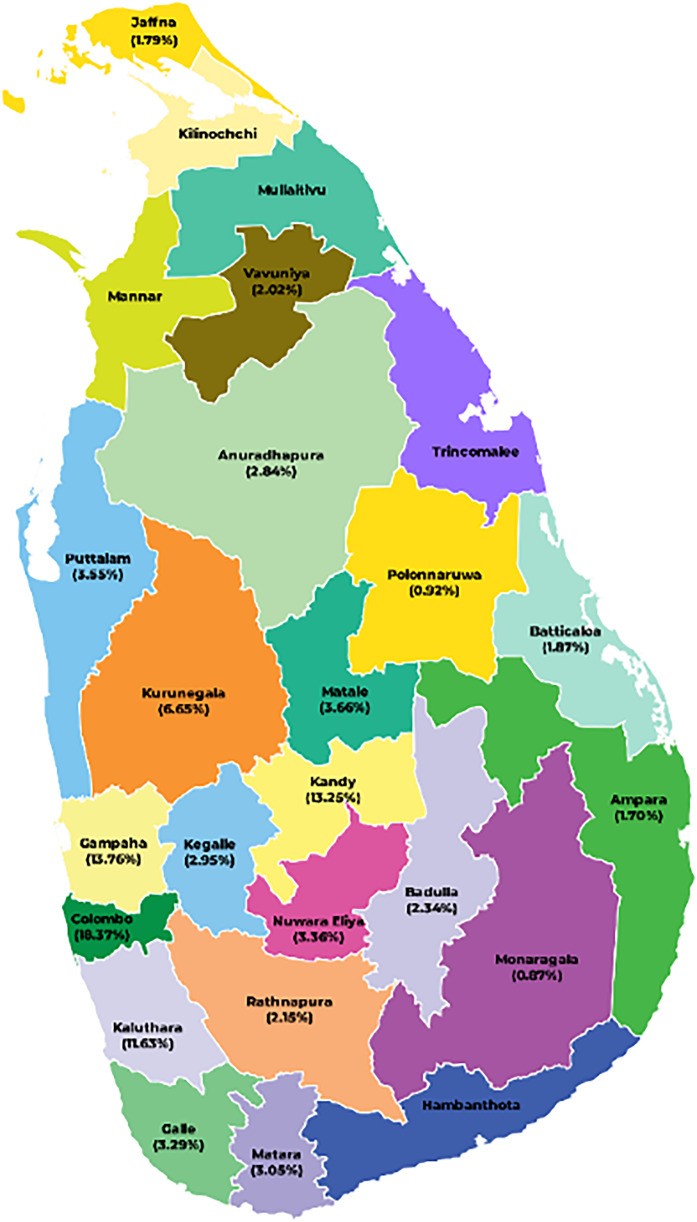
Percentage of the respondents in different districts in Sri Lanka.

**Fig. 2:**
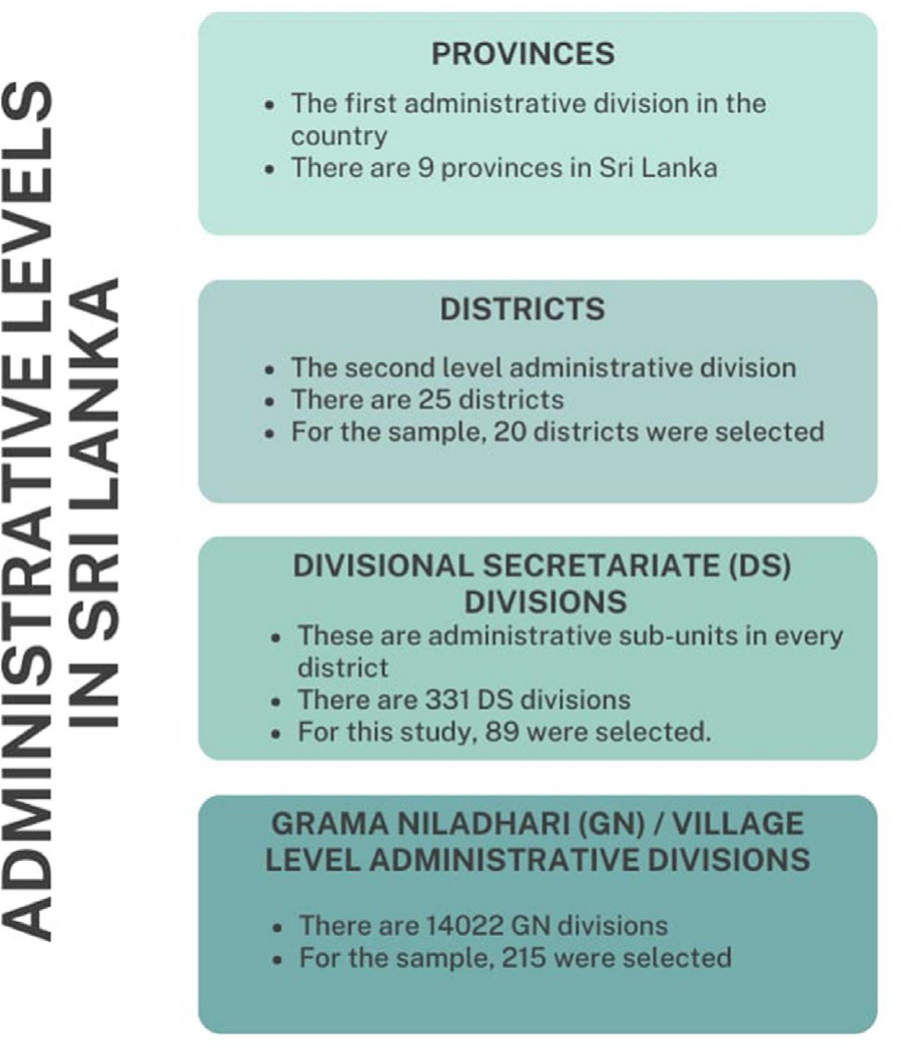
The administrative levels in Sri Lanka.

**Fig. 3:**
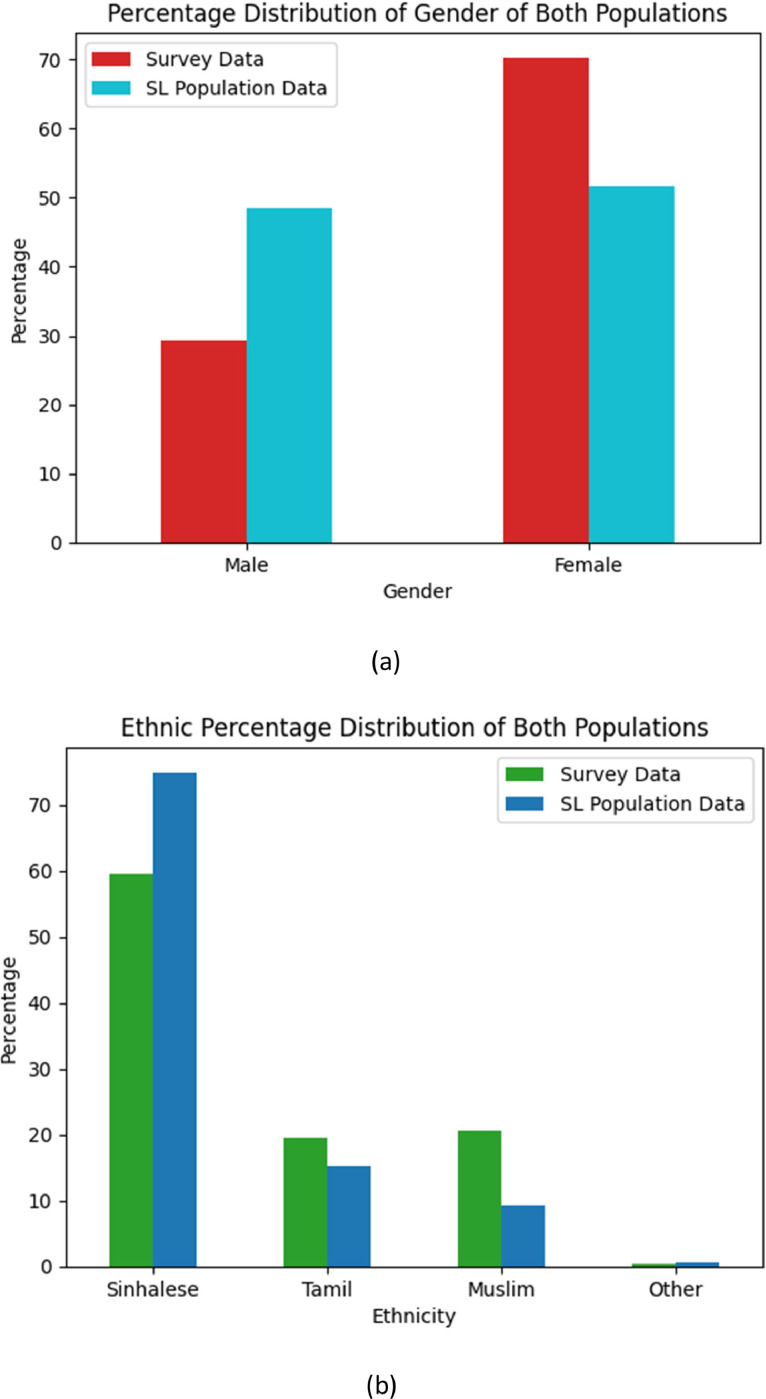
Percentage distribution of the sample demographics of respondents in comparison with the actual population of the country. (a) Gender percentage distribution [2] (b) Ethnic percentage distribution [3] (Please note that the last available government data about the population by gender and ethnicity is from 2021 & 2012 respectively).

Fig. 1 shows the percentage distribution of survey respondents depending on the administrative district they reside. Details about the administrative levels of Sri Lanka are shown in Fig. 2 and described in detail in the Experimental Design, Materials and Methods section. The sample comprises children, youth, adults, and senior citizens. The gender and ethnic identity of the 11,552 individuals from 3020 households are illustrated in Fig. 3. Further, the ethnic distribution of the sample can be justifiable with the actual ethnic distribution of the country (Note that the demographic information included in the data set is related to the main respondent of a family unit for your reference)

## 3 Experimental Design, Materials and Methods

### 3.1 Survey initiation

**Fig. 4:**
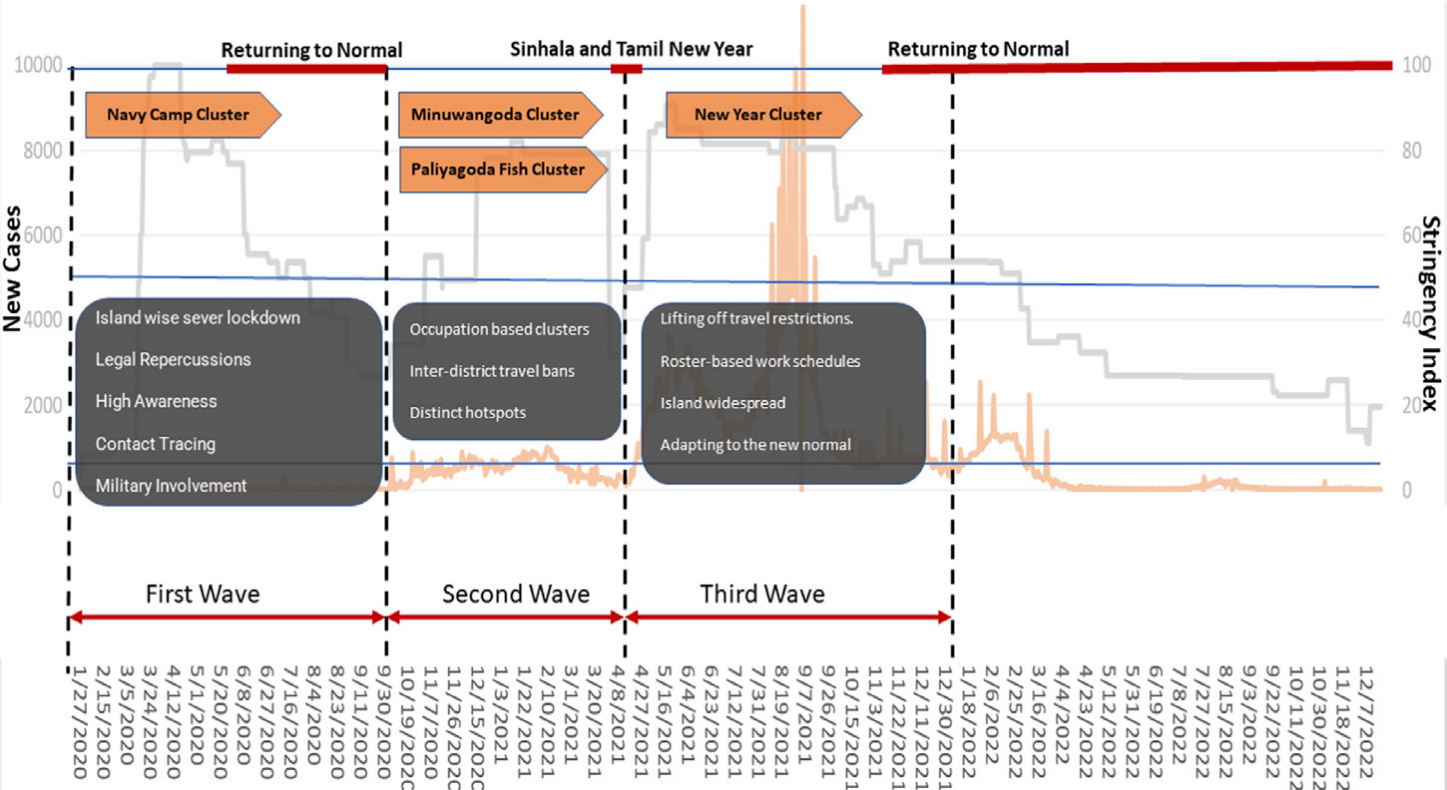
The unique features and labels of the three distinct waves of pandemic.

The survey was initiated on 6th November 2021, two years after the first COVID-19 patient was reported from Sri Lanka [4] and lasted till 10th December 2021. The motivation of capturing the socioeconomic and behavioral impact across the three waves and the pre-pandemic period demands the proper placement of the household survey to capture reliable information when the respondent still can contrast the pandemic waves and the pre-pandemic period. The survey was conducted in the late stage of the third wave when the country was returning to the norm. This placement allowed the authors to capture the detail of the entire pandemic, and the progressive evolution of impacts and attitudes with a thorough wave-wise description from the respondent. This approach of narrowing down the entire pandemic to wave-wise portions while triggering the respondents’ memory using the re-collective tools highlighting unique features of each wave (see Fig. 4), allows the enumerators to capture the respondents’ experiences evolved throughout the pandemic across distinctly identified waves.

### 3.2 Study Instrument

The data collection was conducted through a field household survey by providing an interviewer-administered semi-structured questionnaire that consisted of close-ended and open-ended questions. The questionnaire was modelled to capture different impact areas, namely, 1) Education, 2) Income and economic status, 3) Food consumption, 4) Impact on mobility and human interactions, 5) Health, 6) Cultural, and 7). Psychological. The questionnaire was modeled to capture the impact on these sections throughout the three waves, in contrast to the pre-pandemic period. The timeline of the three waves based on the data from the Epidemiology Unit [5], Ministry of Health, and Sri Lanka is illustrated in Fig. 4. As shown in Table 2, the initial section covers the basic demographic details of the family. The person who responded to this section is considered the main respondent of the survey. The questions related to different impact areas were answered by a relevant person who is associated with the area. (e.g.: the questions on education impact were answered by a member of the family who is engaged in studies.). A Likert scale [6] was used to collect responses for the provided questionnaire. Throughout the questions, three different Likert scales were used to evaluate the level of agreement, level of change, and frequency of attending/visiting. For the level of agreement and level of change, the conventional scale was adapted while a slightly different scale was adapted to capture the frequencies in a probabilistic sense. Here, never is taken as the lower bound and, from there on-wards the scale is built. On a scale of zero to ten, never is taken as zero and the scale is divided into three equal segments to represent the remaining scale (e.g., 1–3.3: less Often, 3.4–6.6: often, 6.7–10: very often). The respondents were informed by the enumerators thoroughly about the scales prior to conducting the interviews. This attempts to accommodate the variance of uncertainty imposed through recollection, using a Likert scale rather than specific numbers. As this was a door-to-door survey the enumerators were given the tools and time to explain these scales and labels, to avoid any confusion.

### 3.3 Selecting the sample

Sri Lanka is a South Asian Island with a 65610 km^2^ area. The country is divided into 9 provinces, which are subdivided into 25 administrative districts. The sample in this study was selected using a multistage clustering technique which was used across the different administrative divisions in Sri Lanka.

Fig. 2. illustrates the top-down administrative structure of Sri Lanka. Further, information regarding the total number of administrative units at each level, as well as the specific number of selected areas, was also provided. Accordingly, this survey was conducted in 202 village-level administrative divisions in the country, distributed over all 9 provinces. Table 3 provides the criteria used to select each administrative division for the multistage clustering. According to Table 3, initially, 20 districts were selected based on the rate of COVID-19 infection. Divisional Secretariats were selected based on the data by the census and statistics department in such a way that it includes regions containing urban areas, rural areas, estate sectors, industrial zones, fisheries zones, agriculture zones, etc. to ensure that the data is unbiased. Furthermore, the poverty head count of the population was also considered to identify the economic condition of the population. Next, the Grama Niladhari (GN) divisions were selected based on the highest proportion of risk due to COVID-19 while accounting for the majority of ethnicity, most of the religions, and minorities included in that GN. The main purpose of the presented data is to analyze the extent of the impact given that a household is affected by the pandemic. The selection of high-risk regions is based on this fact. Finally, households were selected randomly with the help of village-level administrative officers using the lists of registered voters. As a result, the selected households represent diverse groups of individuals across diverse ethnicities, age groups, genders, employment sectors, geographical locations, and other demographic characteristics. A total of 3020 households, with an average of four members in a family, were selected from the village levels randomly to capture the variance in impacts. Fig. 1 illustrates the geographical diversity of the survey. The sample unit of the survey is a single household consisting of responses from several members. The questions related to different impact sections were answered by a relevant member of the household. Finally, all these responses were considered to represent the entire household (a data point). The data set eventually provides a total number of 3020 data points representing the households.

**Table 3:** Criteria followed to select different administrative levels and households.

Administrative Divisions divisions	Criteria to select the divisions	Number of selections at each administrative level
Provinces	All the provinces in Sri Lanka	9
Districts	Considering the severity of the disease spread and containment strategies enforced during the pandemic phases [13]	20
Divisional Secretariate (DS) Divisions	Highly populated DSs from the selected districts [14]	89
Grama Niladhari (GN) divisions	Using the dependency-Ratio (which illustrates the portion of the population over 60 years of age and under 14 years) in the risk map, the Department of Census and Statistics, Sri Lanka [15] High-risk GNs were selected from the DSs. Further, GNs with the following sectors were prioritized; Fisheries, agriculture, estate sector, and industrial areas	202
Households	With the help of respective village-level administrative officers using the list of registered voters.	15 from each GN division

### 3.4 Execution of the field survey

Data collection commenced at the beginning of November 2021 and ended in the mid of December 2021. Computer Assisted Personal Interviews (CAPI) [7] were used for data recording. Enumerators were trained before the survey, which included a role-play through a workshop at the University of Peradeniya, Sri Lanka. All enumerators are social sciences graduates from several state universities in Sri Lanka. Three enumerator teams including a Tamil (the second official language in Sri Lanka) speaking team were engaged in the data collection process. Every enumerator team was under the supervision of an experienced supervisor and a research team member. Enumerators were recruited to overcome religious, cultural, and gender-related barriers during the data collection. Frequent meetings with the enumerators and supervisors were conducted to overcome the obstacles by discussing the challenges and experiences at length throughout the data collection phase. The demographic balance, especially gender balance, among enumerators, was maintained throughout the survey. Each household was interviewed for an average time span of 45 minutes providing a lot of elaboration on the questions to avoid any misunderstandings or confusion. The enumerators used phrases, and examples to trigger the memory of the respondent to get more precise answers. The survey was conducted by taking the household as a unit. Therefore, in the data set a household is taken as a data point. A data point is fully formed from the information gathered through the relevant members of the family. Simultaneously conducting the interview with all the members of a household encouraged the members relevant for each section to answer when needed. For example, a full-time or a part-time student (school student/university student/postgraduate student/diploma student/student engaged in vocational training) was selected to answer the education impact section. The respondent to the questions related to accessing financial localities (second subsection of the mobility impact), was selected based on mostly attending to financial activities. The person who is mainly responsible for buying groceries for the household was selected to answer the mobility section related to food access (the first subsection of the mobility impact). The member of the family who answered the questions on the demographic information on the household is considered the main respondent.

### 3.5 Data reliability through McDonald's omega coefficient

Coefficient omega was proposed 40 years back [8] as a reliability measure of homogeneous items from a measurement instrument. In contrast to a measure like Cronbach's alfa [9], this is a reliability estimate that provides a more accurate estimate of the internal consistency of a multi-item scale [10]. Furthermore, the Omega coefficient can account for multidimensionality, making it a more accurate measure of internal consistency for complex test structures. In literature, McDonald's Omega coefficient has been used to check the reliability of socioeconomic data, including data related to the impact of the COVID-19 pandemic on the economy, education, human mobility [11], psychological behavior [12], and access to health resources. As the overall mobility of a human being depends on multiple indicators such as migration status, length of residence, and travel behavior this measure can be used to evaluate how consistent the indicators are in measuring the underlying construct of mobility [11]. With this light, the McDonald's Omega coefficient is used to evaluate the internal consistency and the reliability of each impact section of the presented data. The results tabulated in Table 4 reflect the technical quality of the data to the user. From Table 4, it can be seen that the omega value of all impact sections is higher than 0.5 (/1), depicting strong reliability. The impact sections covering education, household income, and economy have an Omega coefficient exceeding 0.9. An extended analysis of the reliability of the data related to the income and economy impact section was conducted by extracting the data related to subsection changes in food consumption, resulting in a coefficient value of 0.936. In addition, the data related to mobility, cultural behavior, psychology, and health services and information are within the acceptable range of reliability. Here, the coefficient is calculated for the raw data without conducting any pre-processing. Therefore, high reliability for the above impact section can be achieved by pre-processing the data according to the task of interest.

**Table 4:** McDonald omega coefficient for each impact section.

Impact section	Omega coefficient
Education	0.928
Health Services and information	0.644
Income and economic status	0.936
Mobility	0.572
Psychology	0.644
Cultural	0.528

## Limitations

• It is encouraged to use the data to study the impact of the pandemic at a national level considering that the minimum level of resolution offered for data extraction is a household. The authors discourage using this dataset to conduct quantitative comparisons among different demographic groups, genders, ethnic groups, and geographical regions. For instance, it is not encouraged to use the data to regulate spread, transmission, or relative comparison-based analysis.
• The minimum resolution offered by the dataset is a household. As individual-level information cannot be extracted from the data, individual-level analysis is discouraged.
• As the survey attempts to extract the sense of feelings/perceptions during the identified waves of the pandemic, the provided data relies on the self-reported perception of the ’impact’ of the COVID-19 pandemic. Self-reported data may also be subject to several types of biases, such as social desirability bias or recall bias.
• As the data set is more focused on analyzing the impact of the pandemic, the underlying factors and how deeply the population was affected, the condition to focus on the high-risk regions is vital. Therefore, users should know the demographic distribution of the survey data is conditioned on the highly affected regions, and it won't reflect the actual demographic distribution of the country.

## Ethics Statement

The ethical clearance for this study was obtained from the Ethical Review Committee, Faculty of Arts, University of Peradeniya, Sri Lanka, (ARTS/ERC/2021/01 and 18 September 2021) with the support of the Department of Sociology. Administrative clearance was obtained by the Ministry of Home Affairs of Sri Lanka, relevant DS offices, and relevant GN offices. Informed verbal and written consent were obtained from all the participants. Voluntary participation was ensured. Privacy and confidentiality of data were maintained during each step of data collection, adhering to standard practices and protocols.

## CRediT authorship contribution statement

**Gayanthi A. Ilangarathna:** Conceptualization, Methodology, Writing – original draft. **Lakshitha Ramanayake:** Conceptualization, Methodology, Software, Validation, Writing – original draft. **Neranjan Senarath:** Visualization, Software, Writing – original draft. **Yasiru Ranasinghe:** Visualization, Writing – original draft, Conceptualization, Software. **Harshana Weligampola:** Visualization, Writing – original draft, Software. **Wathsala Dedunupitiya:** Visualization, Writing – original draft. **Isuru Thilakasiri:** Writing – original draft, Visualization. **Roshan Godaliyadda:** Conceptualization, Methodology, Data curation, Supervision, Writing – review & editing. **Parakrama Ekanayake:** Conceptualization, Methodology, Data curation, Supervision, Writing – review & editing. **Vijitha Herath:** Conceptualization, Methodology, Data curation, Supervision, Writing – review & editing. **Janaka Ekanayake:** Project administration, Funding acquisition. **Sakunthala Yatigammana:** Conceptualization, Methodology, Data curation, Supervision. **Anuruddhika Rathnayake:** Writing – review & editing. **Mallika Pinnawala:** Conceptualization, Methodology, Data curation, Supervision. **Muthucumaru Maheswaran:** Data curation. **Ganga Thilakaratne:** Conceptualization, Supervision. **Samath Dharmarathne:** Conceptualization.

## Data Availability

Socioeconomic Impact of COVID-19 during three pandemic waves (Original data) (Dataverse)
